# Lower dietary variety is a relevant factor for malnutrition in older Japanese home-care recipients: a cross-sectional study

**DOI:** 10.1186/s12877-019-1206-z

**Published:** 2019-07-26

**Authors:** Taeko Tsuji, Kaoru Yamamoto, Kazuyo Yamasaki, Fumikazu Hayashi, Chika Momoki, Yoko Yasui, Satoko Ohfuji, Wakaba Fukushima, Daiki Habu

**Affiliations:** 1 Department of Medical Nutrition, Graduate School of Human Life Science, Osaka City University, 3-3-138 Sugimoto, Sumiyoshi-ku, Osaka, 558-8585 Japan; 2 Division of Visiting Nursing, Nishinomiya Social Welfare Corporation, Hyogo, Japan; 3 Office of Epidemiology, Radiation Medical Science Center for the Fukushima Health Management Survey, Fukushima Medical University, Fukushima, Japan; 4 Department of Food and Nutrition, Faculty of Contemporary Human Life Science, Tezukayama University, Nara, Japan; 5 Department of Clinical Nutrition, Graduate School of Human Life Science, Osaka City University, Osaka, Japan; 6 Department of Public Health, Osaka City University Graduate School of Medicine, Osaka, Japan

**Keywords:** Malnutrition, Older people, Home care, Dietary variety, Dietary habits, Dysphagia, Comorbidity

## Abstract

**Background:**

Nutritional status of the older people is affected by various life-style factors. Although dietary habit is one of the life-style factors, it is unknown which of older home-care recipients’ dietary habits are associated with malnutrition.

The purpose of this study was to examine the association of dietary variety, as an evaluation index for dietary habits, with malnutrition in Japanese older home-care recipients.

**Methods:**

This cross-sectional study was conducted in a single city, Hyogo Prefecture, Japan between July and October 2016. Three hundred thirty-three community-dwelling older care recipients (aged 65 years or older who were receiving home-visit nursing care services) were enrolled. Their nutritional status (Mini Nutritional Assessment®-short form: MNA®-SF), dietary variety (Food frequency score [FFS]), socio-demographic characteristics (age, sex, marital status, etc.), health indicators (comorbidity [Charlson Comorbidity Index] and dysphagia status [Dysphagia Severity Scale]) were assessed. The participants were classified into two groups: malnourished (0–7 points) and non-malnourished (8–14 points), according to their MNA®-SF scores. Multivariate logistic regression analysis was used to examine the factors associated with malnutrition.

**Results:**

A total of 317 participants were analyzed (118 men, 199 women, median age: 84 years). Compared to the fourth (highest) quartile of FFS, odds ratios (OR) (95% confidence intervals [CI]) of the third, second, and first (lowest) quartiles of FFS were 1.08 (0.42–2.80), 1.29 (0.56–2.98), and 2.30 (1.02–5.19), respectively (p for trend = 0.049). Higher Charlson Comorbidity Index score and the presence of dysphagia were also significantly associated with malnutrition (OR: 2.08, 95% CI: 1.08–4.00 and OR: 3.86, 95% CI: 2.08–7.17, respectively).

**Conclusion:**

Lower dietary variety was significantly associated with malnutrition in Japanese older home-care recipients.

## Background

With increased life expectancy among the older people, increased health care spending, in particular for institutional care, has become issues of concern in many developed countries [1]. In Japan, the number of home-care recipients has been steadily increasing from 2.65 million in 2007 to 3.85 million in 2017 [2, 3]. The vast majority of home-care recipients are older people [4], and they are provided various home-care services such as home help, home-visit nursing, home-visit rehabilitation, and adult day care. Preventing care needs levels from becoming more severe and prolonging home care may be effective methods of reducing health care costs [1]. Previous studies have reported a negative association between nutritional status and high care level [5, 6]. Other studies have shown a positive correlation between nutritional status and functional capacity in older people [7, 8]. In other words, when the nutritional status of older home-care recipients worsens, there is a possibility of increasing care needs level.

**Table 1:** Participants’ characteristics (*n* = 317)

Sociodemographics	
Age, years	84(77;89)
Female, n(%)	199(62.8)
Living alone, n(%)	90(28.4)
Marital status, n(%)	
Married	163(51.4)
Unmarried	16(5.1)
Divorced/widowed	138(43.5)
Subjective economic status,n(%)	
Excellent	49(16.1)
Good	67(22.0)
Average	157(51.6)
Poor	24(7.9)
Very poor	7(2.3)
Cooking for oneself, n(%)	75(24.0)
Receive Meals on wheels, n(%)	56(17.8)
Health indicators	
Charlson Comorbidity Index, score (*n* = 316)	2.0(1.0;2.5)
DSS	
Within normal limits	228(71.9)
Minimum problems	42(13.3)
Oral problems	17(5.4)
Occasional aspiration	15(4.7)
Water aspiration	15(4.7)
Food aspiration	0(0.0)
Saliva aspiration	0(0.0)
Nutritional status	
BMI, kg/m^2^ (*n* = 267)	21.1(18.6;23.8)
CC, cm (*n* = 282)	30.4(27.5;33.0)
MNA®-SF, score	10.0(8.0;12.0)
Malnourished	59(18.6)
At risk of malnutrition	157(49.5)
Well-nourished	101(31.9)
Dietary variety	
FFS, score	21.0(16.0;23.0)

Data are expressed as the median (25th percentile, 75th percentile) or as number of patients. *DSS* Dysphagia Severity Scale, *BMI* Body Mass Index, *CC* Calf Circumference, *FFS* food frequency score

**Table 2:** Participants’ characteristics for each group

	Malnourished *n* = 59 (18.6%)	Non-malnourished *n* = 258 (81.4%)	*p*-value
Sociodemographics			
Age, years	89(80;93)	83(76;88)	< 0.001
Female, n(%)	40(67.8)	159(61.6)	0.456
Living alone, n(%)	17(28.8)	73(28.3)	1.000
Marital status, n(%)			
Married	24(40.7)	139(53.9)	
Unmarried	1(1.7)	15(5.8)	
Divorced/widowed	34(57.6)	104(40.3)	0.038
Subjective economic status,n(%)			
Excellent	8(14)	41(17)	
Good	10(17.9)	57(23.0)	
Average	30(53.6)	127(51.2)	
Poor	7(12.5)	17(6.9)	
Very poor	1(1.8)	6(2.4)	0.309
Cooking for oneself, n(%)	11(19.3)	64(25.0)	0.397
Receive Meals on wheels, n(%)	12(20.3)	44(17.2)	0.573
Health indicators			
Charlson Comorbidity Index, score	2.0(1.0;3.0)	2.0(1.0;2.0)	0.002
DSS			
Within normal limits	28(47.5)	200(77.5)	
Minimum problems	11(18.6)	31(12.0)	
Oral problems	7(11.9)	10(3.9)	
Occasional aspiration	6(10.2)	9(3.5)	
Water aspiration	7(11.9)	8(3.1)	< 0.001
Dietary variety			
FFS, score	19.0(14.0;23.0)	21.0(17.0;23.0)	0.028

*P*-values from Mann–Whitney U test, Chi-square or Fisher’s exact test. Data are expressed as the median (25th percentile, 75th percentile) or as number of patients. *DSS* Dysphagia Severity Scale, *FFS* food frequency score

**Table 3:** Univariate and multivariate analyses of factors related to malnutrition

	Malnourished (*n* = 59)	Non-malnourished (*n* = 258)	*p*-value^†^	Univariate OR (95%CI)	*p*-value	Multivariate OR (95%CI)	*p*-value
Age, years							
< 84	22(14.4)	131(85.6)		1		1	
≥ 84 (median)	37(22.6)	127(77.4)	0.083	1.7(1–3.10)	0.06	1.44(0.76–2.73)	0.267
Sex							
Male	19(16.1)	99(83.9)		1		1	
Female	40(20.1)	159(79.9)	0.456	1.3(0.7–2.4)	0.38	1.48(0.73–2.98)	0.274
Marital status							
Married	24(14.7)	139(85.3)		1		1	
Others	35(22.7)	119(77.3)	0.083	1.70(1–3)	0.07	1.2(0.60–2.32)	0.627
Charlson Comorbidity Index, score							
< 2	18(12.3)	128(87.7)		1		1	
≥ 2 (median)	41(24.1)	129(75.9)	0.009	2.3(1.2–4.1)	0.008	2.08(1.08–4.00)	0.028
DSS							
Within normal limits (=7)	28(12.3)	200(87.7)		1		1	
Others (< 7)	31(34.8)	58(65.2)	< 0.001	3.8(2.1–6.9)	< 0.001	3.86(2.08–7.17)	< 0.001
FFS, score							
Q4 (Highest: 23–30 points)	15(13.8)	94(86.2)		1		1	
Q3 (21–22 points)	9(16.1)	47(83.9)		1.20(0.5–2.9)	0.69	1.1(0.42–2.80)	0.874
Q2 (16–20 points)	15(19.0)	64(81.0)		1.5(0.7–3.2)	0.34	1.3(0.56–2.98)	0.558
Q1 (Lowest: 0–15 points)	20(27.4)	53(72.6)	0.024	2.4(1.1–5.00)	0.02	2.30(1.02–5.19)	0.044
				(Trend: *p* = 0.025)		(Trend: *p* = 0.049)	

*OR* odds ratio, *CI* confidence interval, *DSS* Dysphagia Severity Scale, *FFS* food frequency score, *Q* quartile

^†^Chi-square or Fisher’s exact test

Malnutrition is a state of nutrition in which a deficiency, excess (or imbalance) of energy, protein and other nutrients causes adverse effects on tissue/body form, function and clinical outcome [9]. Although the term malnutrition can refer to both under- and over-nutrition, it is referred to as undernutrition in this study. Previous studies have reported that various factors associated with hospitalization or nursing home admission in the older people (e.g., multimorbidity, polypharmacy, physical function, and cognitive impairment) [10–13]. Malnutrition is one of them [14, 15]. Therefore, it is important to maintain their good nutritional status as part of medical managements to avoid hospitalization or institutionalization so they can continue in home care. However, malnutrition, or the risk of malnutrition, is prevalent among older home-care recipients in Japan. Approximately 20% of older Japanese home-care recipients are malnourished and more than half of them are at risk for malnutrition [5, 8]. Hence, a strategy for preventing malnutrition among older home-care recipients is an urgent issue in Japan, which is the first nation in the world to experience a super-aged society.

It is difficult for the older people to regain decreased food intake and body weight, because aging may be associated with a significant impairment in the ability to control food intake [16]. In other words, recovering nutritional status is difficult for the already malnourished older people. Therefore, it is important to evaluate the nutritional risk of older home-care recipients early to prevent malnutrition and improve their nutritional status. Nutritional assessment that comprehensively examines nutritional status by using medical history, nutritional history (e.g., appetite, change in food intake, and weigh change), and anthropometric measurements is essential for appropriate nutritional management [17]. In particular, older patients are at nutritional risk, because the physiological changes that occur with advancing age affect nutritional requirements, independent of disease or rehabilitation demands [18]. Thus, their nutritional assessment requires consideration of age and life-style parameters such as physical status, functional status, and socioeconomic factors [18].

Dietary intake is a life-style parameter related to diet. Although the older people who are admitted to the hospital or nursing homes have their diets managed by a food service, the older home-care recipients take their diets freely and individually. Thus, the analysis by trained registered dietitians, who are specialists in nutritional management, is required to assess the actual dietary intake of the older home-care recipients. In Japan, however, a shortage of visiting registered dietitians [19] leads to difficulty in assessing the dietary intake of older home-care recipients in home-care practice. Only a few studies have reported the Japanese older home-care recipients’ dietary intake status such as energy, protein, and other nutrients intake [20, 21]. Additionally, to the best of our knowledge, no published research has referred to the association between their dietary habits and malnutrition, and it is unknown which of their dietary habits are associated with malnutrition.

Dietary variety is one of the convenient evaluation indexes for dietary habits [22, 23]. Previous studies have reported that dietary variety, which was used to assess overall diet quality (i.e., higher dietary variety means well-balanced diet), was associated with all-cause mortality, a decline in high-level functional capacity, physical function, and cognitive function [22, 24–26]. Moreover, having dietary variety is one of the simple ways to promote a balanced intake of nutrients, and such variety may be a good indicator of healthy dietary habits [23].

We hypothesized that we might examine the association between dietary habits with malnutrition in older home-care recipients using dietary variety as an evaluation index for dietary habits. The purpose of this study was to examine the association of dietary variety with malnutrition in older Japanese home-care recipients.

## Methods

### Study design and subjects

This cross-sectional study was conducted in a single city, Hyogo Prefecture, in Japan between July and October 2016. Community-dwelling older care recipients (aged 65 years or older who were receiving home-visit nursing care services provided by four home-visit nursing facilities) were included in this study. The number of the older home-care recipients, who satisfied the inclusion criteria mentioned above during the study period, determined the sample size. Subjects were excluded from the study if they had a medical condition preventing oral feeding, were judged to be unsuitable for this survey by nurses, or were unable to or willing to provide informed consent. Three hundred sixty-eight home-care recipients, who were selected by visiting nurses, were included in this study. All examinations were conducted once per participant by the participants’ regular visiting nurses, who were received nutritional training from a registered dietitian, at the participants’ homes.

### Nutritional assessment

Nutritional status was assessed by the Mini Nutritional Assessment-Short Form (MNA®-SF) [27–30]. The MNA®-SF is one of the most valid and widely used nutritional screening tools for the older people (≥65 years) [31–33]. It consists of six items: decreased food intake in the preceding three months; weight loss during the preceding three months; mobility; psychological stress or acute disease in the preceding three months; neuropsychological problems; and body mass index (BMI) as an anthropometric parameter. The MNA®-SF can substitute calf circumference if BMI is not available. The MNA®-SF classifies individuals as malnourished (0–7 points), at risk of malnutrition (8–11 points), or well-nourished (12–14 points). In this study, the participants were classified into two groups: malnourished (0-7points) and non-malnourished (8-14points), according to their MNA®-SF scores.

### Assessment of dietary variety

There are various ways to assess dietary variety [22–24, 26, 34, 35]. However, because dietary culture varies among countries, it is necessary to use an evaluation method reflecting the dietary habits of Japanese people. Thus, we used the food frequency score (FFS) developed by Kimura et al. [23]. The FFS is known as a simple, valid method of assessing dietary variety for the older people [23, 36]. The FFS evaluates frequency of consumption of 10 main food groups included in Japanese meals (fish and shellfish, meat, eggs, milk, soybean products, dark-colored vegetables, seaweeds, fruits, potatoes, and oils). Food intake frequency was measured using a questionnaire that had 4 choices for each food group: 1) eat almost every day (3 points), 2) eat once every two days (2 points), 3) eat once or twice a week (1 point), and 4) eat hardly ever (0 point). We calculated the FFS as the sum of the scores for each group (range 0–30 points). In the present study, the FFS was evaluated by being classified into one of four levels according to quartiles of FFS: the first quartile (0–15 points, the lowest), the second quartile (16–20 points), the third quartile (21–22 points), and the fourth quartile (23–30 points, the highest).

### Covariates

Socio-demographic characteristics included age, sex, household composition (living alone, or living together), marital status (married, unmarried, or divorced/widowed), subjective economic status (excellent, good, average, poor, or very poor), the person in charge of cooking (oneself or others), and use of Meals on wheels (yes or no). Health indicators included comorbidity and dysphagia status. Comorbidity was evaluated by the Charlson Comorbidity Index [37], which provides a weighted index of comorbidity based on the relative risk of death associated with 19 clinical conditions. There have been reports on the association between malnutrition and comorbidity evaluated using the Charlson Comorbidity Index among older people. [38, 39]. The severity of dysphagia was assessed using the Dysphagia Severity Scale (DSS) [40], a 7-point comprehensive ordinal scale consisting of (1) saliva aspiration, (2) food aspiration, (3) water aspiration, (4) occasional aspiration, (5) oral problems, (6) minimum problems, and (7) within normal limits [40]. A score less than 7 signified the presence of dysphagia, with scores of 1 to 4 being indicative of dysphagia with aspiration [40, 41]. DSS is clinically useful because a videofluoroscopy or videoendoscopic evaluation of swallowing is not always required to determine the severity of dysphagia [42]. The interclass reliability and validity of the DSS have been previously established [41, 43].

### Statistical analysis

To compare the participant characteristics between the malnourished and non-malnourished groups, the Mann-Whitney U test was used for continuous variables, and the Chi-square or Fisher’s exact tests were used for categorical variables.

Univariate and multivariate logistic regression analyses were used to estimate odds ratios (OR) and 95% confidence intervals (CI) for malnutrition (outcome). Independent variables included age (dichotomized at the median), sex, marital status (married vs. other), Charlson Comorbidity Index (dichotomized at the median), DSS (with normal limits vs. other), and FFS. Age, sex, and independent variables with *p*-values less than 0.1 in the univariate analyses, were considered to be potential confounders for adjustment. Statistical tests were considered significant at *p* < 0.05. All statistical analyses were performed using SAS version 9.4 (SAS Institute, Cary, NC, USA).

## Results

From the 368 participants, 35 did not satisfy the following inclusion criteria: aged 65 years or older. Among the remaining 333 participants who answered the questionnaire, MNA®-SF and FFS data were missing for 16 of them. Thus, data from 317 participants were used for this analysis.

Participants’ characteristics are shown in Table 1. The median age was 84 years old, and 62.8% (*n* = 199) of the participants were female. According to MNA®-SF scores, 31.9% (*n* = 101), 49.5% (*n* = 157), and 18.6% (*n* = 59) of all participants were classified as well-nourished, at risk of malnutrition, and malnourished, respectively. Thus, 18.6% (*n* = 59) of the participants were in the malnourished group, while 81.4% (*n* = 258) were in the non-malnourished group.

Table 2 shows the socio-demographic characteristics, health indicators, and dietary variety of both groups. Age and Charlson Comorbidity Index scores were significantly higher in the malnourished than the non-malnourished group (*p* < 0.001 and *p* = 0.002, respectively). On the other hand, FFS was significantly lower in the malnourished group (*p* = 0.028).

Table 3 shows the results of univariate and multivariate logistic regression analyses, which were used to determine the factors associated with malnutrition. Unadjusted univariate logistic regression analysis suggested that three factors, including Charlson Comorbidity Index ≥2, DSS < 7, and the first quartile of FFS, were significantly associated with malnutrition. Furthermore, two factors, including age ≥ 84 and marital status (other than married), tended to be associated with malnutrition. In the multivariate logistic regression analysis which included age, sex, marital status, Charlson Comorbidity Index, DSS, and FFS as explanatory variables, the latter three were found to be independently associated with malnutrition (OR at Charlson Comorbidity Index ≥2: 2.08, 95%CI: 1.08–4.00; OR at DSS < 7: 3.86, 95%CI: 2.08–7.17; and OR at the first [lowest] quartile of FFS: 2.30, 95%CI: 1.02–5.19). With regard to FFS, lower FFS category showed higher OR with a significant dose-response relationship (p for trend = 0.049).

## Discussion

In the present study, we focused on dietary variety as an index of dietary habits and examined the association between dietary habits and malnutrition in older home-care recipients. Logistic regression analyses results revealed that lower dietary variety was independently associated with malnutrition. In other words, the malnourished older home-care recipients might not have taken meals containing a variety of foods. To our knowledge, this is the first study to examine the association of dietary variety with malnutrition in older home-care recipients.

Older home-care recipients need support for many activities related to food intakes, such as buying, cooking, and feeding [44]. However, in the present study, approximately half of the participants in the lowest quartile of FFS were living alone, while those in the highest quartile of FFS were only approximately 20%. Furthermore, although home-care workers play a central role in providing food and nutritional care to the older home-care recipients, they do not have enough time dedicated to food-related care [45]. Therefore, the meals of the older home-care recipients who are living alone may be monotonous.

Although the mechanisms of the association between dietary variety and malnutrition are unclear, it is considered that energy and protein intake was insufficient among the lower dietary variety participants. According to the consensus statement for the identification and documentation of adult malnutrition by the Academy of Nutrition and Dietetics and the American Society for Parenteral and Enteral Nutrition (A.S.P.E.N.), the clinical characteristics of adult malnutrition include decreasing energy intake [46]. In previous reports, there was positive correlation between dietary variety and energy intake among older nursing home residents and community-dwelling older people [34, 47]. In fact, a previous study showed that increasing meal variety increased energy intake in older adults with a poor appetite [48]. Therefore, insufficient energy intake may be a diet-related cause of malnutrition among older home-care recipients with less dietary variety.

On the other hand, Jensen et al. propose an overarching definition of malnutrition as “decline in lean body mass with the potential for functional impairment” [49]. Aging is associated with changes in body composition including reduction of lean body mass; thus, the older people are likely to lose their lean body mass. However, in a previous report, declines in lean body mass were smaller among elderly who had higher protein intake [50]. This suggests that the decline in lean body mass experienced by the elderly is dependent on dietary protein intake. Moreover, previous studies have reported that nutrient intake, including protein, and lean body mass were higher among the elderly with more dietary variety [26, 51]. Consequently, insufficient protein intake may be another diet-related cause of malnutrition in older home-care recipients with less dietary variety.

In the present study, the prevalence of dysphagia and higher comorbidity scores were relevant factors for malnutrition in addition to lower dietary variety. A previous study targeted at older home-care recipients reported that the prevalence of dysphagia was associated with malnutrition [5]. Our result is in line with this finding. Additionally, in a follow-up study targeted at community-dwelling older people, dysphagia was a risk factor for malnutrition [52]. On the other hand, the association between malnutrition and comorbidity is well known, and it has been reported that malnourished older people have higher Charlson Comorbidity Index scores [38, 53]. The International Consensus Committee of A.S.P.E.N. and the European Society for Clinical Nutrition and Metabolism (ESPEN) presented a simple etiology-based construct for the diagnosis of adult malnutrition in a clinical setting [54]. In the commentary, according to the presence and severity of inflammation, adult malnutrition is classified into three categories: “Starvation-related malnutrition,” when there is chronic starvation without inflammation; “Chronic disease-related malnutrition,” when inflammation is chronic and of mild to moderate degree; and “Acute disease or injury-related malnutrition,” when inflammation is acute and of severe degree. The patient who has severe inflammation caused by acute disease or injury needs hospitalization treatment. Hence, our results suggest that the older home-care recipients who have higher Charlson Ccomorbidity Index scores are experiencing chronic disease-related malnutrition. There are many patients with significant medical needs such as chronic illness or dysphagia among the older people receiving home-visit nursing care services. Our results suggest that it is necessary to pay attention to the malnutrition they may also be experiencing.

Our study had several limitations. First, as this was a cross-sectional study, we could not draw a conclusion concerning the causal associations between malnutrition and the relevant factors. Further studies should examine the effectiveness of the food frequency score as a predictor of the older home-care recipients’ nutritional status and prognosis of them in a prospective manner. Second, because the food frequency score assesses overall diet quality by evaluating the frequency of consumption of 10 main food groups, it does not assess actual food intake. Therefore, it was unclear whether the older home-care recipients who had less dietary variety also experienced insufficient energy and nutrient, including protein, intake. Prospective studies that include the quantitative evaluation of food intake are needed.

However, to our knowledge, no other report has examined the association of dietary variety with malnutrition in Japanese older home-care recipients. Because dietary variety is assessed using a questionnaire, any healthcare professional or registered dietitian can assess it easily and objectively. The findings of this study indicate that dietary variety could be a key factor in nutritional assessment and dietary support for older home-care recipients.

## Conclusions

The findings of the present study show lower dietary variety is significantly associated with malnutrition among older Japanese home-care recipients. Prospective intervention studies are required to examine whether nutritional status is improved by increasing dietary variety in older home-care recipients.

## Data Availability

The datasets used and/or analyzed during the current study are available from the corresponding author on reasonable request.

## Abbreviations

95% CI: 95% confidence interval
A.S.P.E.N.: the American Society for Parenteral and Enteral Nutrition
BMI: Body Mass Index
DSS: Dysphagia Severity Scale
ESPEN: the European Society for Clinical Nutrition and Metabolism
FFS: Food frequency score
MNA®-SF: Mini Nutritional Assessment®-short form
OR: Odds ratio
